# Ultra-processed food and beverage advertising on Brazilian television by International Network for Food and Obesity/Non-Communicable Diseases Research, Monitoring and Action Support benchmark

**DOI:** 10.1017/S1368980020000518

**Published:** 2020-10

**Authors:** Julia Soares Guimarães, Laís Amaral Mais, Fernanda Helena Marrocos Leite, Paula Martins Horta, Marina Oliveira Santana, Ana Paula Bortoletto Martins, Rafael Moreira Claro

**Affiliations:** 1 Federal University of Minas Gerais (Universidade Federal de Minas Gerais – UFMG), Department of Nutrition, Belo Horizonte, MG 30130-100, Brazil; 2 Brazilian Institute for Consumer Defense (Instituto Brasileiro de Defesa do Consumidor – IDEC), Department of Healthy and Sustainable Diets, Sao Paulo, Brazil

**Keywords:** Food, Advertising, Television, Surveillance, International Network for Food and Obesity/Non-Communicable Diseases Research, Monitoring and Action Support

## Abstract

**Objective:**

To analyse the extent and nature of food and beverage advertising on the three major Brazilian free-to-air television (TV) channels.

**Design:**

Cross-sectional study. A protocol developed for the International Network for Food and Obesity/Non-Communicable Diseases Research, Monitoring and Action Support was applied for data collection. A total of 432 h of TV programming was recorded from 06.00 to 24.00 hours, for eight non-consecutive and randomly selected days, in April 2018. All TV advertisements (ads) were analysed, and food-related ads were classified according to the NOVA classification system. Descriptive analyses were used to describe the number and type of ads, food categories and the distribution of ads throughout the day and time of the day.

**Setting:**

The three most popular free-to-air channels on Brazilian TV.

**Participants:**

The study did not involve human subjects.

**Results:**

In total, 14·2 % (*n* 1156 out of 7991) of ads were food related (858 were specific food items). Approximately 91 % of food items ads included ultra-processed food (UPF) products. The top three most promoted products were soft drinks, alcoholic beverages and fast-food meals. Alcoholic beverage ads were more frequently broadcast in the evening.

**Conclusion:**

The high risk of exposure of the Brazilian population to UPF ads should be considered a public health concern given the impact of unhealthy food advertising on people’s food choices and health.

High consumption of ultra-processed food (UPF) products^(1,2)^ has been associated with obesity and non-communicable diseases^(1,3)^. Watching television (TV) is an important contributor to those outcomes, through the increase in sedentary activities and influence over food choices^(4,5)^. Further, high-quality scientific evidence highlights the high exposure of different population groups to unhealthy food marketing in TV programming and advertisements (ads)^(6)^. Negative effects related to TV advertising have been observed in both children and adults^(7–9)^. Thus, policy actions to regulate food marketing have become an important part of a set of initiatives to combat obesity and non-communicable diseases^(10,11)^.

Monitoring of food advertising is imperative for the establishment of appropriate measures^(12)^. However, previous studies in the country used low-validity methods to assess this topic, restricting comparability (internationally and nationally – especially over time). Also, this has limited the assessment of the effectiveness of regulatory mechanisms already available in the country such as the Consumer Defense Code^(13)^. The Consumer Defense Code clearly states that ‘all misleading or abusive advertising is prohibited’, including ads of products potentially hazardous to health (such as UPF).

Within this context, the International Network for Food and Obesity/Non-Communicable Diseases Research, Monitoring and Action Support (INFORMAS) has arisen as a global network for public organisations and researchers. The goals of INFORMAS are to monitor, compare and support actions in the public and private sectors, create healthy food environments and prevent obesity and non-communicable diseases^(14)^. Therefore, the aim of this study was to analyse the extent and nature of food and beverage advertising on the three most popular free-to-air channels on Brazilian TV by applying, for the first time, the INFORMAS protocol^(15)^, setting a baseline for food and beverage advertising surveillance in Brazil.

## Methods

This was a cross-sectional study based on the *Food Marketing: Television* protocol developed by INFORMAS^(12,15)^.

The three most popular free-to-air TV channels in Brazil, according to Kantar-IBOPE^(16)^, were included: *Globo*, *Record* and *SBT* (channels account for 90·5 % of total audience – Globo 47·6 %, Record 23·1 % and SBT 19·8 %)^(17)^. Recordings were made for eight non-consecutive days (four weekdays and four weekend days) randomly selected from 1 April to 30 April 2018, from 06.00 to 24.00 hours. The INFORMAS questionnaire was used to catalogue and organise all the information from TV recordings (Epi Info^TM^ version 7.2.2.6)^(18)^. Researchers were trained according to the study protocol. Each food-related advertisement received a unique code (based on product advertised and its duration), while all non-food-related ads received a single generic code. All data extraction was conducted independently by pairs of researchers. All data sets were cross-checked to correct any error (inter-coder reliability = 94·89 %)^(15)^.

For this study, the variables investigated included channel, date of recording, day of the week or weekend, time slot (hour slot, eighteen slots), start and end time of ad, ad type (classified into eight different types as described elsewhere^(15)^), brand/company name, product name and description, and food category (using the NOVA system). A detailed description of the variables collected for each ad can be found elsewhere^(15)^. All food-related ads were classified according to the NOVA classification system^(19,20)^ and linked to nutritional information compiled as part of other research studies^(21)^. In ads with more than one product, the ‘less healthy’ product was selected (based on the NOVA system and the nutrition composition). Food-related ads with no specific product (i.e., brand ads) were not classified. For the analysis, our study units were cluster of ads corresponding to each of the 432 h of recordings (this accounts for the fact that the number of ads may vary according to channel, period of the day and day of the week^(12)^). The absolute and relative frequency of ads belonging to each type (according to the INFORMAS protocol) and to each food group and subgroup was estimated in each study unit. Weighting factors were associated with each unit of study in order to represent exactly the different number of weekdays and weekend hours during the reference period (1–30 April)^(15)^.

Absolute number and the proportion (and respective se and 95 % CI) of each type of ad (on total ads) were estimated. Next, this procedure was repeated for each group and subgroup of food-related ads. Both analyses were conducted for total sample and stratified for weekdays and weekends, and according to the time of the day (morning, afternoon or night). All statistical analysis was conducted using the Stata statistical software package (version 14.2). Differences between values were considered statistically significant when the 95 % CI did not overlap.

## Results

In total, 7991 ads were broadcast over the 432 h of broadcast recorded, at a mean rate of 6·17 ads/channel per hour. Non-food-related ads were the most common type (*n* 6835; 85·8 %; 5·27 ads/channel per hour). A total of 1156 food-related ads (14·2 %) were identified (0·89 ads/channel per hour), of which 922 (11·0 %) belonged to food or drink products (0·71 ads/channel per hour). Food and drink ads were more frequent during the weekend (13·2 %) than weekdays (10·4 %) (Table 1).

**Table 1:** Absolute and relative frequency of ads broadcast on three Brazilian television channels according to the International Network for Food and Obesity/Non-Communicable Diseases Research, Monitoring and Action Support (INFORMAS) classification, by day (weekday *v.* weekend day) (April 2018, *n* 7991)

Advertisement type	*n*	Total			Weekday			Weekend day		
		%	se	95 % CI	%	se	95 % CI	%	se	95 % CI
Food-related advertisements	1156	14·2	0·41	13·4, 15·0	13·8	0·49	12·8, 14·8	15·5	0·64	14·3, 16·8
Food or drink product – food company/brand	922	11·0	0·36	10·3, 11·8	10·4	0·43	9·6, 11·3	13·2	0·60	12·1, 14·5
Food or drink company or brand (no retailer) without food or drink product	21	0·3	0·07	0·2, 0·5	0·4	0·09	0·2, 0·6	0·1	0·06	0·0, 0·3
Food or drink retailer (supermarket or convenience store) with food or drink product	40	0·6	0·10	0·4, 0·8	0·8	0·12	0·5, 1·0	0·1	0·06	0·0, 0·3
Food or drink retailer (supermarket or convenience store) without food or drink product	59	0·7	0·09	0·5, 0·9	0·6	0·10	0·4, 0·8	1·0	0·18	0·7, 1·4
Food or drink retailer (restaurant or takeaway or fast food) with food or drink product	94	1·2	0·13	1·0, 1·5	1·3	0·16	1·0, 1·7	0·9	0·17	0·6, 1·3
Food or drink retailer (restaurant or takeaway or fast food) without food or drink product	20	0·3	0·06	0·2, 0·4	0·3	0·08	0·2, 0·5	0·2	0·07	0·1, 0·4
Non-food-related advertisements	6835	85·8	0·41	85·0, 86·6	86·2	0·49	85·2, 87·2	84·5	0·64	83·2, 85·7
Total	7991	100·0			100·0			100·0		

Nine out of ten food and drink ads (90·8 %) included at least one UPF (0·60 ads/channel per hour). Soft drinks (28·9 %, 0·19 ads/channel per hour), alcoholic beverages (14·3 %, 0·10 ads/channel per hour) and fast-food meals (13·8 %, 0·08 ads/channel per hour) were the top three most promoted products, representing more than half of all food and drink ads. The proportion of UPF ads did not vary between weekdays and weekends (Table 2).

**Table 2:** Frequency of food or drink product ads according to the NOVA classification system, by day (weekday *v* weekend days) (April 2018, *n* 858)*

Food product category	Total				Weekday				Weekend			
	*n*	%	se	95 % CI	*n*	%	se	95 % CI	*n*	%	se	95 % CI
Unprocessed and minimally processed foods	67	7·6	0·96	5·9, 9·7	37	7·4	1·17	5·4, 10·1	30	8·4	1·47	5·9, 11·7
Coffee	26	2·8	0·59	1·9, 4·3	13	2·6	0·71	1·5, 4·4	13	3·6	0·99	2·1, 6·2
Fresh meat	24	2·5	0·55	1·6, 3·9	11	2·2	0·66	1·2, 3·9	13	3·6	0·99	2·1, 6·2
Fresh milk and milk products	8	0·9	0·33	0·4, 1·8	4	0·8	0·39	0·3, 2·1	4	1·1	0·56	0·4, 2·9
Others	9	1·4	0·46	0·7, 2·6	9	1·8	0·60	0·9, 3·4	0	†		
Processed culinary ingredients	7	1·0	0·37	0·5, 2·1	6	1·2	0·48	0·5, 2·6	1	0·3	0·28	0·0, 2·0
Processed foods	4	0·6	0·30	0·2, 1·6	4	0·8	0·39	0·3, 2·1	0	†		
Ultra-processed food and drink products	780	90·8	1·06	88·5, 92·6	453	90·6	1·31	87·7, 92·9	327	91·3	1·49	87·9, 93·8
Soft drinks	246	28·9	1·65	25·8, 32·2	146	29·2	2·04	25·4, 33·3	100	27·9	2·37	23·5, 32·8
Alcoholic beverages	133	14·3	1·24	12·0, 16·8	63	12·6	1·49	10·0 –15·8	70	19·5	2·10	15·7, 24·0
Fast-food meals	109	13·8	1·28	11·5, 16·5	76	15·2	1·61	12·3, 18·6	33	9·2	1·53	6·6, 12·7
Nuggets and other ultra-processed meat products	83	10·0	1·10	8·0, 12·4	52	10·4	1·37	8·0, 13·4	31	8·7	1·49	6·1, 12·1
Ice-cream, chocolate and candies	57	6·5	0·89	5·0, 8·5	32	6·4	1·10	4·6, 8·9	25	7·0	1·35	4·7, 10·1
Other sweetened beverages	53	5·8	0·83	4·3, 7·6	26	5·2	0·99	3·6, 7·5	27	7·5	1·40	5·2, 10·8
Pastries, cakes and cookies	37	4·4	0·76	3·2, 6·2	23	4·6	0·93	3·1, 6·8	14	3·9	1·03	2·3, 6·5
Margarines	35	3·7	0·66	2·6, 5·2	16	3·2	0·79	2·0, 5·2	19	5·3	1·19	3·4, 8·2
Sauces	15	1·9	0·50	1·1, 3·1	10	2·0	0·63	1·1, 3·7	5	1·4	0·62	0·6, 3·3
Savoury packaged snacks	8	1·0	0·38	0·5, 2·1	6	1·2	0·49	0·5, 2·6	2	0·6	0·39	0·1, 2·2
Others	4	0·5	0·27	0·2, 1·5	3	0·6	0·35	0·2, 1·9	1	0·3	0·28	0·0, 2·0
Total	858	100·0			500	100·0			358	100·0		

* Condiments, sugar, sweeteners, oils and fats, classified as processed culinary ingredients and processed foods (salted, cured or smoked meats) had a low *n* value when compared with other categories of the NOVA system.

† No cases.

When comparing the frequency of food or drink ads by the time of the day (Table 3), unprocessed and minimally processed food ads were broadcast more in the morning than in the afternoon or evening (15·4 *v.* 3·6 % and 15·4 *v*. 6·7 %, respectively). However, no difference was found for UPF ads. When comparing the NOVA subgroups, alcoholic beverages were more commonly promoted in the evening than in the morning or afternoon (21·6 *v.* 8·3 % and 21·6 *v.* 8·6 %, respectively).

**Table 3:** Frequency of food or drink product ads according to NOVA classification system by time of the day (morning, afternoon and evening) (April 2018, *n* 858)*

Food product category	Morning				Afternoon				Evening			
	*n*	%	se	95 % CI	*n*	%	se	95 % CI	*n*	%	se	95 % CI
Unprocessed and minimally processed foods	28	15·4	2·75	10·7, 21·7	16	3·6	0·98	2·1, 6·1	23	6·7	1·48	4·3, 10·3
Coffee	11	6·7	1·96	3·8, 11·8	8	1·5	0·56	0·7, 3·1	7	1·6	0·65	0·7, 3·5
Fresh meat	8	3·5	1·32	1·7, 7·3	4	1·1	0·57	0·4, 3·0	12	3·6	1·11	1·9, 6·5
Fresh milk and milk products	1	0·3	0·26	0·0, 1·9	4	1·1	0·57	0·4, 3·0	3	1·1	0·66	0·3, 3·5
Others	8	4·9	1·69	2·5, 9·5	0	†			1	0·4	0·44	0·1, 3·1
Processed culinary ingredients	1	0·6	0·61	0·1, 4·3	3	1·1	0·64	0·4, 3·4	3	1·1	0·66	0·3, 3·5
Processed foods	0	†			0	†			4	1·8	0·89	0·7, 4·7
Ultra-processed food and drink products	175	83·9	2·80	77·6, 88·7	348	95·2	1·16	92·4, 97·1	257	90·4	1·80	86·2, 93·4
Soft drinks	66	32·8	3·50	26·3, 40·0	118	32·6	2·64	27·7, 38·0	62	21·6	2·58	16·9, 27·1
Fast-food meals	32	17·2	2·86	12·3, 23·6	55	16·1	2·10	12·4, 20·7	22	8·6	1·80	5·6, 12·9
Alcoholic beverages	25	8·3	1·77	5·4, 12·5	46	11·7	1·76	8·6, 15·6	62	21·6	2·58	17·0, 27·1
Nuggets and other ultra-processed meat products	15	7·8	2·02	4·6, 12·8	31	8·8	1·61	6·1, 12·6	37	13·0	2·11	9·3, 17·7
Other sweetened beverages	16	7·4	1·90	4·4, 12·1	25	5·9	1·26	3·9, 9·0	12	4·3	1·29	2·4, 7·7
Pastries, cakes and cookies	9	4·5	1·53	2·2, 8·7	18	5·5	1·32	3·7, 8·7	10	3·2	1·07	1·6, 6·1
Margarines	6	3·3	1·37	1·5, 7·4	19	4·8	1·16	2·9, 7·6	10	2·7	0·92	1·4, 5·2
Ice-cream, chocolate and candies	6	2·6	1·15	1·1, 6·1	24	6·6	1·40	4·3, 10·0	27	9·3	1·81	6·3, 13·5
Sauces	0	†			9	2·5	0·89	1·2, 5·0	6	2·4	1·00	1·1, 5·4
Savoury packaged snacks	0	†			3	0·7	0·44	0·2, 2·4	5	2·2	0·99	0·9, 5·3
Others	0	†			0	†			4	1·5	0·79	0·5, 4·2

* Condiments, sugar, sweeteners, oils and fats, classified as processed culinary ingredients and processed foods (salted, cured or smoked meats) had a low *n* value when compared with other categories of the NOVA system.

† No cases.

## Discussion

The systematic recording of the three most popular free-to-air TV channels in Brazil, following the INFORMAS protocol, allowed thorough analysis of the extent and nature of food and beverage advertising in the country. Even though this is not the first study on this topic in Brazil^(22,23)^, it innovates by being the first one based on an international protocol, which reinforces its validity, lays the foundation for future national studies and enables comparability with results from other countries^(6,15)^. There are two other studies similar to this one, the first one was conducted in 2009^(22)^ and the second in 2014^(23)^. The first analysed the programming of the three most popular free-to-air TV channels in Brazil during ten consecutive days from 08.00 to 18.00 hours^(22)^, and the second analysed the programming of the four most popular free-to-air TV channels in Brazil, but during only two non-consecutive days from 06.30 to 23.00 hours^(23)^. In the first case, the concentration of recordings over a short period could compromise the external validity of the results, and the peak audience hours (from 20.00 to 22.30 hours) were not included. In the second, the recordings were spread over an insufficient period and number of days to capture the variability of the ads. Finally, both studies also used different systems to classify advertising (according to the number of groups that food and non-food-related ads should be classified into), since no protocol was available at the time, restricting the validity of the comparison with future studies. These differences reinforced the importance of a benchmark protocol. It is noteworthy that all these weaknesses were addressed and overcome with the protocol adopted by the present study. Similar scenarios to those observed in the present study were found in both investigations: the proportion of food-related ads remaining between 10 and 15 % (13·8 % in 2009, 10·2 % in 2014 and 11·0 % in 2018) and sugar-sweetened beverages and alcoholic beverages figuring among the most broadcasted food-related ads^(22,23)^. However, time-trend conclusions should be avoided, since the validity of these initial findings is potentially low and agreement analysis between the values will not lead to meaningful conclusions.

Currently, twenty-two countries have their TV food advertising benchmark data collected under the same methods applied in this study^(6)^. The similarities with our results and those countries’ data reveal the major aspect of food patterns globalisation based on the increasing expansion of the food industry, especially of big transnational food corporations^(24–26)^. Although food advertising varies among countries, UPF always have a prominent position^(6)^. Even in countries with contrasting dietary pattern, like Malaysia^(27)^ and Argentina^(28)^, UPF ads represented most of the food-related ads (70·0 and 95·3 %, respectively), and sugar-sweetened beverages and fast-food meals/restaurants were the most broadcasted products.

Although our results do not allow direct identification of population exposure to unhealthy food advertising, considering that the Brazilian population report watching TV for at least 3 h/d^(29,30)^ and the average rate of UPF ads (0·60 ads/channel per hour), an average Brazilian would be exposed to 657 UPF ads/year. As life expectancy in the country is 76 years^(31)^, supposing constant exposure to TV, in a lifetime, an average Brazilian would be exposed to 49 932 UPF ads. This is concerning since it is known that both long and acute exposure to unhealthy food ads (e.g., during a 40-min TV programme or a 5-min advergame) can affect food choices among all age groups^(32)^. Globally, policy discussions relating to food marketing are focused on children rather than the adult population. Unfortunately, detailed information on age group audience is not publicly available for Brazil. However, evidence suggests that child audiences follow peak viewing patterns. Since children and adolescents in Brazil attend school part-time (part at morning or part during afternoon), the higher concentration of children at home is limited to the evening. Thus, almost no shows dedicated to children are available at free-to-air channels (mostly restricted to a few hours in one channel in Saturday mornings)^(33–35)^. As a result, a survey conducted by Kantar Media IBOPE already indicated that soap operas, series and live soccer games were the three most watched programmes by the audience between 4 and 17 years old^(36)^, all of which are broadcasted in the evening (from 19.00 to 24.00 hours)^(33–35)^.

Some limitations should be considered. Our sample only included free-to-air TV channels and the year of 2018 may have been an atypical year for marketing due to the Soccer World Cup. However, we considered that those had minor impact on the results. Free-to-air TV is still the main source of information and entertainment in Brazil, reaching 93 % of the national population^(16)^ and while the World Cup may have affected ad content, it seems less likely that this type of event impacts the profile of products advertised (for what can be seen in other countries using the same method^(6)^). However, only through continuous monitoring, it will be possible to accurately estimate the impact of special events on food advertising.

Our results reinforce the need to monitor and regulate food advertising on TV. Actions to stimulate healthy eating behaviours and to reduce overweight and non-communicable diseases will be more effective once the food environment contributes to it, which involves reducing unhealthy food advertising exposure^(11)^. Currently, Brazil already has advertising regulations in place, especially the Consumer Defense Code^(13)^. Although the Code has been available since 1990, the lack of specific criteria for its application in relation to advertising regulation is (as our results make clear) compromising its effectiveness and should be soon reviewed.

Finally, one of the main objectives of the INFORMAS monitoring is to provide information to support policies and to substantiate government’s decision^(12,14)^. Thus, this article looks forward to laying the foundation through our results for effective regulations against the exposure to unhealthy food advertising, as well as actions to promote consumers’ empowerment (i.e., sharing data related to this issue in public schools, health centres and all kinds of media) to identify and combat unhealthy food advertising.
